# Supplementary material for: Therapeutic efficacy of dimethyl fumarate in relapsing-remitting multiple sclerosis associates with ROS pathway in monocytes
Source: Nat Commun. 2019 Jul 12;10:3081. doi: 10.1038/s41467-019-11139-3 (PMC6626021; doi:10.1038/s41467-019-11139-3)
Supplement: Supplementary file 1 — Supplementary Information [file 41467_2019_11139_MOESM1_ESM.pdf]

Therapeutic efficacy of dimethyl fumarate in relapsing-remitting multiple sclerosis associates with ROS pathway in monocytes

Carlström et al

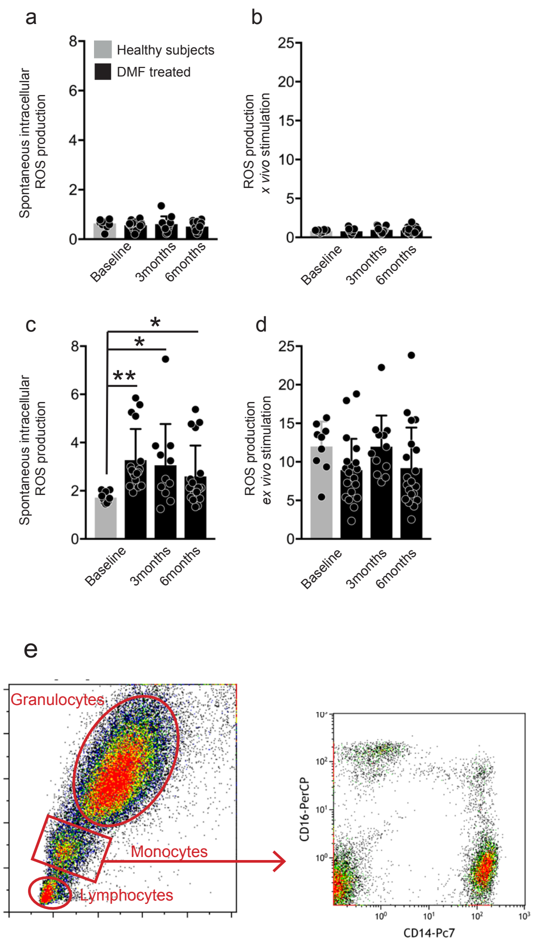

Supplementary Fig. 1. ROS generation in granulocytes and lymphocytes. (a, b) ROS generation in lymphocytes from healthy subjects or DMF
treated patients. (c, d) ROS generation in granulocytes from healthy subjects or DMF treated patients. (e) Sequential gating stratiergy. Graphs show means and S.D.. All analysis between healthy controls and patients
are performed with Welch's t test. *P<0.05, **P<0.01

Supplementary Table 1. Canonical pathways affected by differentially expressed genes in CD14+ monocytes after 6 months of treatment with DMF.

Ingenuity Canonical Pathway	-LOG(p-val)	Ratio	Molecules	z-score
Protein Ubiquitination Pathway	3,42	0,042	UBE2D4,PSMD10,DNAJC5,CUL2,PSMB2,BAG1,PSMA5,DNAJC10,UBE2E3,NEDD4L,ANAPC11	NaN
Thiosulfate Disproportionation III (Rhodanese)	3,37	0,667	MOCS3,TST	NaN
Mechanisms of Viral Exit from Host Cells	2,84	0,098	VPS25,ACTA2,LMNB1,TSG101	NaN
NRF2-mediated Oxidative Stress Response	2,63	0,042	MGST1,DNAJC5,NRAS,GAB1,ACTA2,DNAJC10,UBE2E3,TXN	NaN
EIF2 Signaling	2,27	0,036	RPL4,NRAS,RPL18A,GAB1,ACTA2,RPL17,RPS25,RPS2	1,342
FAK Signaling	2,17	0,051	NRAS,CAPN11,GAB1,ACTA2,PTEN	NaN
Oxidative Phosphorylation	1,99	0,046	COX8A,SURF1,NDUFAB1,ATP5MC3,NDUFA8	2,236
Hypoxia Signaling in the Cardiovascular System	1,90	0,053	UBE2D4,UBE2E3,LDHA,PTEN	NaN
Role of Macrophages, Fibroblasts and Endothelial Cells in Rheumatoid Arthritis	1,87	0,029	WNT3A,NRAS,GAB1,CHP1,TLR8,FZD2,FCGR1A,FCGR3A/FCGR3B,PRSS1	NaN
Systemic Lupus Erythematosus Signaling	1,70	0,031	SNRPN,LSM6,NRAS,CD40,GAB1,FCGR1A,FCGR3A/FCGR3B	NaN
NF-kB Signaling	1,65	0,033	CSNK2A2,NRAS,CD40,GAB1,TLR8,TBK1	2,449
UDP-D-xylose and UDP-D-glucuronate Biosynthesis	1,62	0,500	UGDH	NaN
β-alanine Degradation I	1,62	0,500	ALDH6A1	NaN
Role of NFAT in Regulation of the Immune Response	1,60	0,032	NRAS,GAB1,CHP1,FCGR1A,FCGR3A/FCGR3B,GNG7	1,342
Fcγ Receptor-mediated Phagocytosis in Macrophages and Monocytes	1,59	0,043	ACTA2,FCGR1A,FCGR3A/FCGR3B,PTEN	1
Fatty Acid α-oxidation	1,55	0,091	TMLHE,ALDH2	NaN
Melanoma Signaling	1,55	0,055	NRAS,GAB1,PTEN	NaN
D-myo-inositol (1,4,5,6)-Tetrakisphosphate Biosynthesis	1,54	0,035	PPP1R7,PPTC7,PPP4R1,PPP4C,PTEN	2,236
D-myo-inositol (3,4,5,6)-tetrakisphosphate Biosynthesis	1,54	0,035	PPP1R7,PPTC7,PPP4R1,PPP4C,PTEN	2,236
Prostate Cancer Signaling	1,53	0,041	NRAS,GAB1,NKX3-1,PTEN	NaN
Hereditary Breast Cancer Signaling	1,52	0,035	NRAS,GAB1,RFC2,POLR2J,PTEN	NaN
Ovarian Cancer Signaling	1,52	0,035	WNT3A,NRAS,GAB1,FZD2,PTEN	NaN
Colorectal Cancer Metastasis Signaling	1,52	0,028	WNT3A,NRAS,GAB1,TLR8,FZD2,PTGER4,GNG7	1,89
Huntington's Disease Signaling	1,50	0,028	NSF,DNAJC5,CAPN11,GAB1,POLR2J,GNG7,NAPB	NaN
Phagosome Maturation	1,48	0,034	NSF,VPS18,ATP6V1C1,TSG101,NAPB	NaN
3-phosphoinositide Biosynthesis	1,46	0,030	GAB1,PPP1R7,PPTC7,PPP4R1,PPP4C,PTEN	2,449
L-carnitine Biosynthesis	1,45	0,333	TMLHE	NaN
Mouse Embryonic Stem Cell Pluripotency	1,41	0,038	WNT3A,NRAS,GAB1,FZD2	1
3-phosphoinositide Degradation	1,39	0,032	PPP1R7,PPTC7,PPP4R1,PPP4C,PTEN	2,236
Endometrial Cancer Signaling	1,38	0,047	NRAS,GAB1,PTEN	NaN
IL-2 Signaling	1,38	0,047	CSNK2A2,NRAS,GAB1	NaN
D-myo-inositol-5-phosphate Metabolism	1,35	0,031	PPP1R7,PPTC7,PPP4R1,PPP4C,PTEN	2,236
Glioblastoma Multiforme Signaling	1,34	0,031	WNT3A,NRAS,GAB1,FZD2,PTEN	1
Phenylethylamine Degradation I	1,33	0,250	ALDH2	NaN
Molybdenum Cofactor Biosynthesis	1,33	0,250	MOCS3	NaN
Paxillin Signaling	1,33	0,035	ITGA2B,NRAS,GAB1,ACTA2	NaN
Integrin Signaling	1,31	0,027	ITGA2B,NRAS,CAPN11,GAB1,ACTA2,PTEN	1,633

Supplementary Table 1. Canonical pathways affected by differentially expressed genes in CD14+ monocytes after 6 months of treatment with DMF.

DMF: Dimethyl fumarate.
Input gene list was based on differentially expressed genes with p-value < 0.01 and averageRMA > 4.
The table includes all canonical pathways from Ingenuity Pathway Analysis with Fisher's p-value < 0.05.
z-score: the activation z-score is used to infer likely activation states of upstream regulators (z-score > 2 correspods to activation).

Supplementary Table 2. Canonical pathways affected by differentially methylated genes in CD14+ monocytes after treatment with DN

Ingenuity Canonical Pathway	-LOG(p-val)	Ratio	Molecules	N mol	Group	Cluster	ROS Score
Growth Hormone Signaling	4,15	0,104	PIK3R5,IGF1R,IRS2,RPS6KA2,STAT3,PRKCZ,RPS6KC1,PRKCB	8	M3 vs. M6	2	0
FLT3 Signaling in Hematopoietic Progenitor Cells	3,99	0,099	PIK3R5,STAT2,IRS2,MAPK13,RPS6KA2,STAT3,CREB5,INPP5D	8	M3 vs. M6	1	0
Melanocyte Development and Pigmentation Signaling	2,93	0,078	GNAS,PRKAG2,PIK3R5,IRS2,RPS6KA2,CREB5,RPS6KC1	7	M3 vs. M6	1	0
Dopamine-DARPP32 Feedback in cAMP Signaling	2,90	0,062	PRKG1,GNAS,PPP1R1B,CSNK1G3,PPM1L,PRKAG2,CREB5,PRKCZ,PRKCB	9	M3 vs. M6	3	0
mTOR Signaling	2,72	0,054	PPM1L,PRKAG2,PIK3R5,RPTOR,IRS2,RPS6KA2,PRKCZ,PLD1,RPS6KC1,PRKCB	10	M3 vs. M6	1	0
Tec Kinase Signaling	2,70	0,058	LCK,GNAS,GNA15,PIK3R5,STAT2,IRS2,STAT3,PRKCZ,PRKCB	9	M3 vs. M6	2	0
IL-15 Signaling	2,67	0,081	LCK,PIK3R5,IRS2,IL6,MAPK13,STAT3	6	M3 vs. M6	1	0,17
IL-3 Signaling	2,47	0,074	PIK3R5,IRS2,STAT3,PRKCZ,INPP5D,PRKCB	6	M3 vs. M6	2	0
Gap Junction Signaling	2,45	0,053	PRKG1,GNAS,CSNK1G3,PRKAG2,PIK3R5,IRS2,PRKCZ,GJC1,PRKCB	9	M3 vs. M6	3	0
Adrenomedullin signaling pathway	2,43	0,053	PPARG,PRKG1,C3,GNAS,GNA15,PRKAG2,PIK3R5,IRS2,MAPK13	9	M3 vs. M6	1	0
Renin-Angiotensin Signaling	2,43	0,064	PRKAG2,PIK3R5,IRS2,MAPK13,STAT3,PRKCZ,PRKCB	7	M3 vs. M6	2	0
HGF Signaling	2,39	0,063	DOCK1,PIK3R5,IRS2,IL6,STAT3,PRKCZ,PRKCB	7	M3 vs. M6	2	0,14
Synaptic Long Term Depression	2,36	0,056	PRKG1,GNAS,GNA15,GRID2,PPM1L,IGF1R,PRKCZ,PRKCB	8	M3 vs. M6	3	0
PDGF Signaling	2,34	0,070	PIK3R5,IRS2,STAT3,INPP5D,PRKCB,PDGFRB	6	M3 vs. M6	1	0
Axonal Guidance Signaling	2,30	0,038	GLI2,GNAS,PIK3R5,EPHA3,PRKCZ,ADAMTS2,DOCK1,EPHA6,GNA15,GLIS1,PRKAG2,NFATC2,BMP7,IR	15	M3 vs. M6	1	0
Superpathway of Inositol Phosphate Compounds	2,29	0,047	ITPK1,MINPP1,LCK,TNS3,PPP1R1B,PPM1H,PIK3R5,IRS2,INPP5D,PDGFRB	10	M3 vs. M6	1	0
GÎ±q Signaling	2,29	0,054	GNAS,GNA15,PIK3R5,NFATC2,IRS2,PRKCZ,PLD1,PRKCB	8	M3 vs. M6	2	0
Calcium-induced T Lymphocyte Apoptosis	2,29	0,081	LCK,NFATC2,PRKCZ,ORAI1,PRKCB	5	M3 vs. M6	3	0
NGF Signaling	2,28	0,060	PIK3R5,TRIO,IRS2,RPS6KA2,CREB5,PRKCZ,RPS6KC1	7	M3 vs. M6	1	0
eNOS Signaling	2,27	0,054	AQP5,PRKG1,GNAS,PRKAG2,PIK3R5,IRS2,PRKCZ,PRKCB	8	M3 vs. M6	2	0
Thrombopoietin Signaling	2,26	0,079	PIK3R5,IRS2,STAT3,PRKCZ,PRKCB	5	M3 vs. M6	2	0
UVB-Induced MAPK Signaling	2,26	0,079	PIK3R5,IRS2,MAPK13,PRKCZ,PRKCB	5	M3 vs. M6	2	0
Corticotropin Releasing Hormone Signaling	2,24	0,059	GLI2,GNAS,PRKAG2,MAPK13,CREB5,PRKCZ,PRKCB	7	M3 vs. M6	3	0
CREB Signaling in Neurons	2,23	0,049	GNAS,GNA15,GRID2,PRKAG2,PIK3R5,IRS2,CREB5,PRKCZ,PRKCB	9	M3 vs. M6	3	0
ERK/MAPK Signaling	2,21	0,049	PPARG,DOCK1,PPM1L,PRKAG2,PIK3R5,IRS2,STAT3,CREB5,PRKCB	9	M3 vs. M6	1	0
3-phosphoinositide Biosynthesis	2,21	0,049	ITPK1,MINPP1,LCK,TNS3,PPP1R1B,PPM1H,PIK3R5,IRS2,PDGFRB	9	M3 vs. M6	1	0
IL-12 Signaling and Production in Macrophages	2,13	0,056	PPARG,PIK3R5,IRS2,MAPK13,PRKCZ,PRKCB,APOD	7	M3 vs. M6	2	0
Role of NFAT in Cardiac Hypertrophy	2,11	0,047	GNAS,PRKAG2,PIK3R5,IGF1R,IRS2,IL6,MAPK13,PRKCZ,PRKCB	9	M3 vs. M6	2	0,11
LXR/RXR Activation	2,07	0,061	ECHS1,C3,NR1H3,IL6,IL1RAPL1,APOD	6	M3 vs. M6	1	0,33
Insulin Receptor Signaling	2,04	0,054	ASIC2,PRKAG2,PIK3R5,RPTOR,IRS2,PRKCZ,INPP5D	7	M3 vs. M6	1	0
IGF-1 Signaling	2,03	0,060	PRKAG2,PIK3R5,IGF1R,IRS2,STAT3,PRKCZ	6	M3 vs. M6	2	0
Nitric Oxide Signaling in the Cardiovascular System	2,01	0,059	PRKG1,PRKAG2,PIK3R5,IRS2,PRKCZ,PRKCB	6	M3 vs. M6	2	0
Endothelin-1 Signaling	1,98	0,048	GNAS,GNA15,PIK3R5,IRS2,MAPK13,PRKCZ,PLD1,PRKCB	8	M3 vs. M6	2	0
IL-17A Signaling in Airway Cells	1,95	0,067	PIK3R5,IRS2,IL6,MAPK13,STAT3	5	M3 vs. M6	1	0,20
FGF Signaling	1,90	0,065	PIK3R5,IRS2,MAPK13,STAT3,CREB5	5	M3 vs. M6	1	0
Role of NFAT in Regulation of the Immune Response	1,87	0,046	LCK,GNAS,GNA15,CSNK1G3,PIK3R5,NFATC2,IRS2,ORAI1	8	M3 vs. M6	1	0
Thrombin Signaling	1,87	0,046	GNAS,GNA15,PIK3R5,IRS2,MAPK13,ARHGEF10,PRKCZ,PRKCB	8	M3 vs. M6	2	0
3-phosphoinositide Degradation	1,86	0,050	ITPK1,MINPP1,TNS3,PPP1R1B,PPM1H,INPP5D,MTMR3	7	M3 vs. M6	1	0
Glioma Signaling	1,84	0,055	PIK3R5,IGF1R,IRS2,PRKCZ,PRKCB,PDGFRB	6	M3 vs. M6	2	0
CXCR4 Signaling	1,84	0,049	DOCK1,GNAS,GNA15,PIK3R5,IRS2,PRKCZ,PRKCB	7	M3 vs. M6	2	0
JAK/Stat Signaling	1,81	0,062	PIK3R5,STAT2,IRS2,IL6,STAT3	5	M3 vs. M6	1	0,20
Prolactin Signaling	1,81	0,062	PIK3R5,IRS2,STAT3,PRKCZ,PRKCB	5	M3 vs. M6	2	0
GNRH Signaling	1,81	0,049	GNAS,GNA15,PRKAG2,MAPK13,CREB5,PRKCZ,PRKCB	7	M3 vs. M6	3	0
Role of Tissue Factor in Cancer	1,80	0,054	LCK,GNA15,PIK3R5,IRS2,MAPK13,RPS6KA2	6	M3 vs. M6	1	0
Natural Killer Cell Signaling	1,80	0,054	LCK,PIK3R5,IRS2,PRKCZ,INPP5D,PRKCB	6	M3 vs. M6	2	0
Fc Epsilon RI Signaling	1,79	0,053	PIK3R5,IRS2,MAPK13,PRKCZ,INPP5D,PRKCB	6	M3 vs. M6	2	0
NF-kB Activation by Viruses	1,73	0,059	LCK,PIK3R5,IRS2,PRKCZ,PRKCB	5	M3 vs. M6	2	0
LPS-stimulated MAPK Signaling	1,73	0,059	PIK3R5,IRS2,MAPK13,PRKCZ,PRKCB	5	M3 vs. M6	2	0
GPCR-Mediated Nutrient Sensing in Enteroendocrine Cells	1,73	0,059	GNAS,GNA15,PRKAG2,PRKCZ,PRKCB	5	M3 vs. M6	3	0
B Cell Receptor Signaling	1,72	0,043	APBB1IP,PIK3R5,NFATC2,IRS2,MAPK13,CREB5,INPP5D,PRKCB	8	M3 vs. M6	1	0
CCR3 Signaling in Eosinophils	1,72	0,051	GNAS,PIK3R5,IRS2,MAPK13,PRKCZ,PRKCB	6	M3 vs. M6	2	0
Breast Cancer Regulation by Stathmin1	1,71	0,043	GNAS,PPM1L,PRKAG2,PIK3R5,IRS2,ARHGEF10,PRKCZ,PRKCB	8	M3 vs. M6	2	0
Aldosterone Signaling in Epithelial Cells	1,71	0,046	ASIC2,DNAJC5,DNAJC8,PIK3R5,IRS2,PRKCZ,PRKCB	7	M3 vs. M6	2	0
PAK Signaling	1,69	0,058	ARHGAP10,PIK3R5,IRS2,EPHA3,PDGFRB	5	M3 vs. M6	1	0
fMLP Signaling in Neutrophils	1,69	0,050	GNAS,PIK3R5,NFATC2,IRS2,PRKCZ,PRKCB	6	M3 vs. M6	2	0
IL-6 Signaling	1,67	0,050	PIK3R5,IRS2,IL6,IL1RAPL1,MAPK13,STAT3	6	M3 vs. M6	1	0,17
CDK5 Signaling	1,67	0,057	GNAS,PPP1R1B,PPM1L,PRKAG2,MAPK13	5	M3 vs. M6	3	0
Adrenomedullin signaling pathway	1,66	0,029	C3,MAPK14,KCNQ2,PIK3R5,KCNN2	5	BL vs. M3	1	0,20
p70S6K Signaling	1,66	0,050	PPM1L,PIK3R5,IRS2,PRKCZ,PLD1,PRKCB	6	M3 vs. M6	2	0
P2Y Purigenic Receptor Signaling Pathway	1,64	0,049	PRKAG2,PIK3R5,IRS2,CREB5,PRKCZ,PRKCB	6	M3 vs. M6	2	0
ErbB Signaling	1,62	0,055	PIK3R5,IRS2,MAPK13,PRKCZ,PRKCB	5	M3 vs. M6	2	0
PI3K Signaling in B Lymphocytes	1,61	0,048	C3,NFATC2,IRS2,PRKCZ,INPP5D,PRKCB	6	M3 vs. M6	3	0
AMPK Signaling	1,60	0,041	GNAS,PPM1L,PRKAG2,PIK3R5,RPTOR,IRS2,MAPK13,CREB5	8	M3 vs. M6	1	0
Fcγ Receptor-mediated Phagocytosis in Macrophages and Monoc	1,60	0,054	DOCK1,PRKCZ,PLD1,INPP5D,PRKCB	5	M3 vs. M6	3	0
Huntington's Disease Signaling	1,59	0,039	DNAJC5,GNA15,PIK3R5,IGF1R,DNM3,IRS2,CREB5,PRKCZ,PRKCB	9	M3 vs. M6	1	0
Role of Pattern Recognition Receptors in Recognition of Bacteria	1,55	0,047	C3,PIK3R5,IRS2,IL6,PRKCZ,PRKCB	6	M3 vs. M6	2	0,17
Cardiac Hypertrophy Signaling	1,50	0,039	GNAS,GNA15,PRKAG2,PIK3R5,IGF1R,IRS2,IL6,MAPK13	8	M3 vs. M6	1	0,13
UVA-Induced MAPK Signaling	1,50	0,051	PIK3R5,IRS2,MAPK13,RPS6KA2,RPS6KC1	5	M3 vs. M6	1	0
G Beta Gamma Signaling	1,50	0,051	GNAS,GNA15,PRKAG2,PRKCZ,PRKCB	5	M3 vs. M6	3	0
Type II Diabetes Mellitus Signaling	1,49	0,046	PPARG,PRKAG2,PIK3R5,IRS2,PRKCZ,PRKCB	6	M3 vs. M6	2	0
Xenobiotic Metabolism Signaling	1,48	0,037	AHRR,PPM1L,PIK3R5,IRS2,IL6,MAPK13,CYP2B6,PRKCZ,PRKCB	9	M3 vs. M6	1	0,11
G-Protein Coupled Receptor Signaling	1,45	0,036	GNAS,GNA15,HTR7,PRKAG2,PIK3R5,IRS2,STAT3,CREB5,PRKCB	9	M3 vs. M6	1	0
NF-kB Signaling	1,45	0,041	LCK,PIK3R5,IGF1R,IRS2,PRKCZ,PRKCB,PDGFRB	7	M3 vs. M6	2	0
Opioid Signaling Pathway	1,43	0,038	LCK,GNAS,PRKAG2,GRK5,RPS6KA2,CREB5,PRKCZ,PRKCB	8	M3 vs. M6	3	0
Paxillin Signaling	1,42	0,049	DOCK1,ACTN2,PIK3R5,IRS2,MAPK13	5	M3 vs. M6	1	0
Neuropathic Pain Signaling In Dorsal Horn Neurons	1,42	0,049	PRKAG2,PIK3R5,IRS2,PRKCZ,PRKCB	5	M3 vs. M6	2	0
Phospholipase C Signaling	1,42	0,038	LCK,GNAS,NFATC2,CREB5,ARHGEF10,PRKCZ,PLD1,PRKCB	8	M3 vs. M6	3	0
Relaxin Signaling	1,41	0,044	GNAS,GNA15,PRKAG2,PIK3R5,IRS2,PRKCZ	6	M3 vs. M6	2	0
Production of Nitric Oxide and Reactive Oxygen Species in Macro	1,40	0,040	PPM1L,PIK3R5,IRS2,MAPK13,PRKCZ,PRKCB,APOD	7	M3 vs. M6	2	0
Sperm Motility	1,40	0,048	PRKG1,GNAS,PRKAG2,PRKCZ,PRKCB	5	M3 vs. M6	3	0
Signaling by Rho Family GTPases	1,37	0,037	GNAS,CDH18,GNA15,PIK3R5,IRS2,ARHGEF10,PRKCZ,PLD1	8	M3 vs. M6	1	0
Glucocorticoid Receptor Signaling	1,35	0,033	PRKAG2,PIK3R5,NFATC2,TAT,IRS2,IL6,MAPK13,STAT3,KRT20,TSC22D3	10	M3 vs. M6	1	0,10
Systemic Lupus Erythematosus Signaling	1,35	0,039	SNRPN,LCK,PIK3R5,NFATC2,IRS2,IL6,INPP5D	7	M3 vs. M6	1	0,14
Hepatic Cholestasis	1,34	0,042	PRKAG2,NR1H3,IL6,IL1RAPL1,PRKCZ,PRKCB	6	M3 vs. M6	3	0,17
p38 MAPK Signaling	1,33	0,046	IL1RAPL1,MAPK13,RPS6KA2,CREB5,RPS6KC1	5	M3 vs. M6	1	0
Synaptic Long Term Potentiation	1,31	0,046	GNA15,PRKAG2,CREB5,PRKCZ,PRKCB	5	M3 vs. M6	3	0

Supplementary Table 2. Canonical pathways affected by differentially methylated genes in CD14+ monocytes after treatment with DMF.

DMF: Dimethyl fumarate; BL: baseline; M3: 3 months after initation of DMF treatment; M6: 6 months after initation of DMF treatment.
Input gene list was based on genes associated with differentially methylated probes that displayed delta b > 5% methylation change and p-value < 0.001.
The table includes all canonical pathways from Ingenuity Pathway Analysis with Fisher's p-value < 0.05 and number of molecules ≥ 5

ROS score indicates % overlap with a list of assembled known ROS genes:

ACADL	CD83	ELK1	HADHB	IL6	MYC	SDHB	TNC
ACADM	CD84	EPHA2	HBEGF	INHBA	MYD88	SDHC	TNFAIP8
ACADS	CDC14C	ETFDH	HCAR2	IRAK1	NAIP	SDHD	TNFRSF11A
ACADVL	CDC45	FADD	HGF	IRAK4	NCF2	SERPINA1	TNS1
ACAT1	CDHR1	FAR1	HIF1A	IRF1	NCF4	SHC1	TOLLIP
ACO1	CDKN1A	FAR2	HIF1A-AS1	IRF2	NFE2L2	SHC3	TPI1
ACO2	CDKN1B	FAR2P2	HLA-A	IRF3	NFE2L2	SIRPA	TRAF2
ACOT9	CDKN2B	FCGR1A	HLA-B	IRF9	NFIX	SIRPD	TRAJ22
ACSL1	CES1	FCGR3B	HLA-C	ITGA2	NFKB1	SLC25A20	TRAJ30
ACSL3	CES2	FCGRT	HLA-DOA	ITGA7	NKAPL	SLC27A2	TRAV13-1
ACSL4	CES3	FDXR	HLA-DRB1	ITGAX	NOD1	SLC7A11	TRAV38-1
ACSL5	CES4A	FGF13	HLA-F	ITGB4	NOG	SMAD1	TRAV4
ACSL6	CES5A	FH	HLA-L	JUN	NOS2	SMAD2	TRBJ2-3
ADH7	CLEC12B	FMO5	HMGCL	JUNB	NOX1	SMAD3	TRBV5-1
AGER	CLEC4A	FN1	HMGCS2	KLHDC10	NOX3	SMAD5	TRGV5
ALDH3A1	CLEC4C	FOS	HMOX1	KLHL1	NOX4	SMAD7	TXN2
APOBEC1	CLEC4E	FOXP3	HSP90AA1	LAX1	NOX5	SMAD9	TXNRD1
APOL3	CLEC5A	FPR1	HSPA1A	LCNL1	NOXA1	SOD1	TXNRD2
APOOL	CLEC6A	FPR2	ICAM1	LOC100287651	NOXO1	SOD2	TXNRD3
APP	CLEC7A	FPR3	IDH2	LPL	NPL	SOD3	UGT1A1
ARHGEF18	CLUH	FST	IDH3A	LTBP1	NQO1	SORBS1	VASP
ARHGEF7	COL1A2	FTL	IDH3B	LTBP2	OGDH	SOS1	VAV1
ATG4A	CPT1A	FYN	IDH3G	LTBP2	OLR1	SP1	VAV2
BCL2	CPT2	G6PD	IFI27L1	LY6G6D	OXCT1	SQSTM1	VAV3
BDH1	CRAT	GAS2L3	IFNA1	LY6H	PC	SRXN1	VEGFA
BLVRB	CS	GBP1	IFNAR1	LY9	PCK2	SSTR3	XDH
BMP4	CSF2	GCDH	IFNAR2	MAFF	PDCD7	STAT1	ZYX
BRAF	CTH	GCLC	IFNB1	MAFG	PDGFB	SUCLG1	
CAMK2N1	CXCL10	GCLM	IFNG	MAL	PDHA1	SUCLG2	
CAPNS1	CXCL11	GGT1	IFNG-AS1	MAOA	PDP2	TAP1	
CASP8	CXCL9	GK2	IFNGR1	MAP2K3	PDPR	TBK1	
CAT	CYBA	GPD2	IFNGR2	MAP2K6	PECR	TBRG4	
CAV1	CYBB	GPX1	IGDCC4	MAPK12	PGD	TCL1B	
CAV2	CYP1A1	GPX2	IGHD	MAPK14	POLDIP2	TERT	
CAV3	CYP2A6	GPX3	IGKV1D-39	MAPK15	PPARD	TGFA	
CBR1	CYP4A11	GRB2	IGKV1D-8	MAPK8	PRDX1	TGFB1	
CBR3	DECR1	GSR	IGKV1OR-3	MDFI	PRDX6	TGFB2	
CBS	DLD	GSTA1	IGLV8-61	MDH2	PREX1	TGFB2-AS1	
CCL5	DLST	GSTA2	IL10	ME1	PSMB9	TGFBR1	
CCND1	DNAJB1	GSTA3	IL10RB	MET	PTGER1	TGFBR2	
CCR6	DOCK2	GSTA4	IL12A	MGST1	PTGR1	TGFBR3	
CD11c	DUOX1	GSTM2	IL12B	MGST3	PTK2	THBS1	
CD14	DUOX2	GSTM3	IL15	MKI67	PXN	TIAM2	
CD19	DUOXA1	GSTM4	IL17A	MLYCD	RAC1	TIRAP	
CD1E	DUOXA2	GSTM5	IL17D	MMP1	RAC2	TLR2	
CD38	ECHS1	GSTP1	IL17RE	MMP12	SCP2	TLR4	
CD40	ECI1	GSTT1	IL19	MPST	SDHAF2	TLR5	
CD47	EGR1	GSTT2	IL1A	MST1R	SDHAP1	TLR6	
CD5	EHHADH	HADH	IL1B	MT1X	SDHAP2	TLR9	
CD69	EIF2AK2	HADHA	IL23A	MTFR1	SDHAP3	TMEM8B	

Supplementary Table 3. Canonical pathways affected by differentially methylated genes in CD4+ T-cells after treatment with DMF.

Ingenuit	-LOG(p-val)	Ratio	Molecul	N mol	Group	Cluster	ROS Score
1D-myo-i	1,43	0,294	ITPK1,ITP	5	BL vs. M6	1	0
3-phosph	2,10	0,190	FYN,PTPN	35	M3 vs. M	4	0,06
3-phosph	1,77	0,168	G6PC2,NU	31	BL vs. M6	1	0,03
3-phosph	2,26	0,204	PTPN2,NU	29	M3 vs. M	4	0
3-phosph	2,58	0,197	G6PC2,NU	28	BL vs. M6	1	0
Amyloid F	1,83	0,234	PRKACB,A	11	BL vs. M6	2	0,18
Androgen	1,49	0,174	PRKACB,C	21	BL vs. M6	2	0,14
Apoptosis	1,48	0,183	GAS2,MA	17	BL vs. M6	3	0,29
ATM Sign	2,64	0,237	MAP2K4,I	22	M3 vs. M	5	0
Axonal Gu	1,40	0,157	PRKACB,K	62	M3 vs. M	5	0,08
Axonal Gu	1,72	0,149	DPYSL2,P	59	BL vs. M6	3	0,05
B Cell Rec	1,68	0,166	MAP2K6,I	31	BL vs. M6	3	0,26
BMP signa	1,41	0,189	PRKACB,M	14	BL vs. M6	2	0,43
Calcium S	1,66	0,183	PRKACB,A	30	M3 vs. M	5	0
Calcium S	2,50	0,189	PRKACB,A	31	BL vs. M6	2	0
Calcium-i	1,34	0,194	CALM1 (ir	12	BL vs. M6	2	0,08
cAMP-me	1,75	0,179	AKAP12,P	36	M3 vs. M	5	0
cAMP-me	3,52	0,199	AKAP12,P	40	BL vs. M6	2	0
Cardiac Î²	3,04	0,231	AKAP12,P	28	M3 vs. M	5	0
Cardiac Î²	3,73	0,231	AKAP12,P	28	BL vs. M6	2	0
CD28 Sign	1,34	0,181	MAP2K4,I	23	M3 vs. M	5	0,09
CDK5 Sign	2,23	0,223	ITGB1,PRI	21	M3 vs. M	5	0
CDK5 Sign	2,04	0,202	PRKACB,P	19	BL vs. M6	2	0,05
Colorecta	1,33	0,026	JAK1,GAB	6	BL vs. M3	7	0
Corticotro	1,39	0,185	PRKACB,A	22	M3 vs. M	5	0
Corticotro	1,83	0,185	NOS1,PRK	22	BL vs. M6	2	0
CXCR4 Sig	1,59	0,034	DOCK1,PA	5	BL vs. M3	7	0
D-myo-in	1,43	0,294	ITPKB,INP	5	BL vs. M6	1	0
D-myo-in	2,29	0,209	PTPN2,NU	27	M3 vs. M	4	0
D-myo-in	2,27	0,194	G6PC2,NU	25	BL vs. M6	1	0
D-myo-in	2,29	0,209	PTPN2,NU	27	M3 vs. M	4	0
D-myo-in	2,27	0,194	G6PC2,NU	25	BL vs. M6	1	0
D-myo-in	2,17	0,201	PTPN2,NU	29	M3 vs. M	4	0
D-myo-in	2,20	0,188	G6PC2,NU	27	BL vs. M6	1	0
Dopamine	1,43	0,209	PRKACB,A	14	M3 vs. M	5	0
Dopamine	3,92	0,284	PRKACB,P	19	BL vs. M6	2	0,05
Dopamine	2,13	0,200	PRKACB,A	29	M3 vs. M	5	0
Dopamine	3,41	0,214	NOS1,PRK	31	BL vs. M6	2	0
Ephrin Re	1,42	0,030	PAK1,EPH	5	BL vs. M3	6	0
Epithelial	1,83	0,197	TCF4,ARP	25	M3 vs. M	5	0,04
Epithelial	1,78	0,181	AKT2,TCF	23	BL vs. M6	3	0,13
Fc Epsilon	1,31	0,168	MAP2K6,I	20	BL vs. M6	3	0,15
FcÎ³RIIB Si	1,50	0,194	MAP2K4,(	14	BL vs. M6	3	0,07
FXR/RXR	2,37	0,053	MLXIPL,SI	5	BL vs. M3	6	0
G Beta Ga	1,73	0,202	PRKACB,A	21	M3 vs. M	5	0,05
G Beta Ga	2,20	0,202	PRKACB,A	21	BL vs. M6	2	0,05
GABA Rec	3,55	0,286	ADCY2,UE	20	M3 vs. M	5	0
GÎ±12/13	1,91	0,041	CDH18,GA	5	BL vs. M3	7	0
GÎ±i Signa	1,71	0,185	PRKACB,A	20	BL vs. M6	2	0,05
GÎ±q Sign	1,35	0,163	AKT2,HTR	24	BL vs. M6	3	0,13
GNRH Sig	1,57	0,033	PAK1,MA	5	BL vs. M3	7	0
GNRH Sig	1,46	0,180	MAP2K6,I	27	M3 vs. M	5	0,15
GNRH Sig	1,48	0,167	MAP2K6,I	25	BL vs. M6	2	0,28
GP6 Signa	1,96	0,042	ITGA2B,G	5	BL vs. M3	6	0
GP6 Signa	3,10	0,218	COL4A3,C	26	BL vs. M6	3	0,15
GPCR-Me	1,53	0,188	PRKACB,A	16	BL vs. M6	2	0
Hepatic C	1,68	0,174	MAP2K4,I	25	BL vs. M6	2	0,20
Hereditar	1,54	0,184	NPM1,AR	26	M3 vs. M	5	0
HGF Signa	2,71	0,052	DOCK1,PA	6	BL vs. M3	7	0
HIPPO sig	1,36	0,198	SMAD2,YA	16	M3 vs. M	5	0,25
HIPPO sig	2,80	0,235	SMAD2,P	19	BL vs. M6	3	0,21
Huntingto	2,02	0,167	MAP2K4,A	39	BL vs. M6	3	0,05
Î±-Adrene	1,33	0,181	PRKACB,A	15	BL vs. M6	2	0
IL-15 Proc	3,07	0,375	PTK2,PRK	9	BL vs. M6	3	1
IL-15 Sign	1,45	0,188	IL15RA,AI	15	BL vs. M6	3	0,47
IL-17A Sig	1,68	0,200	MAP2K4,A	15	BL vs. M6	3	0,33
IL-22 Sign	1,83	0,292	MAP2K4,A	7	BL vs. M6	3	0,14
IL-6 Signa	1,32	0,167	MAP2K6,I	21	BL vs. M6	3	0,29
ILK Signal	1,41	0,030	DOCK1,GA	5	BL vs. M3	7	0
Insulin Re	1,46	0,169	PRKACB,P	23	BL vs. M6	2	0,04
Leukocyte	1,46	0,159	MAP2K4,I	31	BL vs. M6	3	0,23
Melatonin	2,60	0,242	MAP2K6,I	16	BL vs. M6	2	0,06
Molecular	2,13	0,171	MAP2K4,I	62	M3 vs. M	5	0,21
Molecular	1,94	0,154	MAP2K4,I	56	BL vs. M6	3	0,21
Netrin Sig	3,88	0,327	PRKACB,F	17	M3 vs. M	5	0
Netrin Sig	3,84	0,308	PRKACB,F	16	BL vs. M6	2	0
Neuroinfl	2,14	0,178	MAP2K4,A	49	M3 vs. M	5	0,24
Neuroinfl	1,52	0,153	MAP2K4,I	42	BL vs. M6	3	0,38
Nitric Oxi	1,72	0,188	PRKACB,A	19	BL vs. M6	2	0,05
nNOS Sign	3,04	0,295	NOS1,CAF	13	BL vs. M6	2	0
nNOS Sign	2,03	0,300	SNTB2,RY	9	M3 vs. M	5	0
nNOS Sign	1,78	0,267	NOS1,CAI	8	BL vs. M6	2	0
Opioid Sig	2,63	0,195	MAP2K6,I	42	M3 vs. M	5	0,07
Opioid Sig	2,92	0,186	MAP2K6,I	40	BL vs. M6	2	0,10
Paxillin Si	2,12	0,046	DOCK1,PA	5	BL vs. M3	7	0
Phototran	2,15	0,270	PRKACB,G	10	BL vs. M6	2	0
PI3K/AKT	1,91	0,041	JAK1,FOX	5	BL vs. M3	7	0,20
PI3K/AKT	1,73	0,195	ITGB1,GA	24	M3 vs. M	5	0,08
PPARÎ±/R	1,34	0,160	MAP2K6,I	26	BL vs. M6	2	0,31
Productio	1,80	0,170	MAP2K4,A	30	BL vs. M6	3	0,20
Protein Ki	2,36	0,176	PRKACB,P	60	M3 vs. M	5	0,03
Protein Ki	2,93	0,170	PRKACB,P	58	BL vs. M6	2	0,09
PTEN Sign	1,94	0,203	ITGB1,MA	24	M3 vs. M	5	0,17
PXR/RXR	2,41	0,255	PRKACB,A	13	BL vs. M6	2	0,08
Rac Signa	1,46	0,188	ITGB1,MA	22	M3 vs. M	5	0,27
Rac Signa	2,21	0,197	MAP2K4,T	23	BL vs. M6	3	0,48
RAR Activ	1,97	0,175	MAP2K4,I	30	BL vs. M6	2	0,27
Relaxin Si	1,40	0,167	PRKACB,A	23	BL vs. M6	2	0,13
Renin-Ang	1,67	0,181	MAP2K4,I	21	BL vs. M6	2	0,38
Role of BF	2,64	0,250	IFNG,RBL	19	M3 vs. M	5	0,05
Role of BF	1,33	0,184	RBL2,RBB	14	BL vs. M6	3	0
Role of Cl	1,77	0,236	RPA1,PPP	13	M3 vs. M	5	0
Role of JA	1,73	0,280	MAP2K4,T	7	BL vs. M6	3	0,29
Role of JA	2,27	0,348	IFNG,PTPI	8	M3 vs. M	5	0,13
Role of NI	3,71	0,203	MAP2K6,I	40	BL vs. M6	2	0,08
Role of O:	1,99	0,033	FOXO1,GA	7	BL vs. M3	7	0
Role of PK	1,41	0,220	MAP2K6,T	9	BL vs. M6	3	0,89
Sertoli Ce	2,08	0,180	MAP2K4,I	29	BL vs. M6	3	0,07
Signaling	1,45	0,028	PAK1,CDH	6	BL vs. M3	7	0
Sonic Hec	2,07	0,296	PRKACB,P	8	BL vs. M6	2	0
Sperm Mo	1,73	0,202	PNPLA8,P	21	M3 vs. M	5	0,10
Sperm Mo	1,33	0,173	PRKACB,G	18	BL vs. M6	2	0,11
Superpatl	1,49	0,273	IMPAD1,I	6	BL vs. M6	1	0
Superpatl	1,62	0,175	FYN,PTPN	37	M3 vs. M	4	0,05
Superpatl	1,60	0,160	G6PC2,NU	34	BL vs. M6	1	0,03
Synaptic I	3,44	0,228	GRID2,PL	34	M3 vs. M	5	0
Synaptic I	1,75	0,174	NOS1,GRI	26	BL vs. M6	2	0
Synaptic I	1,75	0,184	PRKACB,P	21	BL vs. M6	2	0
Tec Kinas	1,52	0,032	PAK1,JAK	5	BL vs. M3	7	0
Telomera	1,79	0,202	HDAC9,HI	22	M3 vs. M	5	0,05
Tight Junc	1,60	0,173	PRKACB,C	24	BL vs. M6	2	0,13
Type I Dia	2,19	0,048	JAK1,HLA	5	BL vs. M3	7	0,20
Type II Di	1,78	0,038	GAB1,SH2	5	BL vs. M3	7	0
Type II Di	1,36	0,167	MAP2K4,A	22	BL vs. M6	3	0,36

Supplementary Table 3. Canonical pathways affected by differentially methylated genes in CD4+ T-cells after treatment with DMF.

DMF: Dimethyl fumarate; BL: baseline; M3: 3 months after initation of DMF treatment; M6: 6 months after initation of DMF treatment.

Input gene list was based on genes associated with differentially methylated probes that displayed delta b > 5% methylation change and p-value < 0.001.

The table includes all canonical pathways from Ingenuity Pathway Analysis with Fisher's p-value < 0.05 and number of molecules ≥ 5.

Supplementary Table 4. Functions affected by differentially methylated genes in CD4+ T-cells after treatment with DMF.

Categories	Diseases or Functions Annotation	P-val	Molecules	N mol	Group
Hematological System Development and Function, Tissue Morpho	Quantity of mononuclear leukocytes	3,90E-02	CDK6,CHST2,CXCL	18	BL vs. M3
Cell Death and Survival	Apoptosis of B lymphocytes	1,65E-02	BCL11A,BCL2A1,B	26	BL vs. M6
Cell Death and Survival	Apoptosis of B-lymphocyte derived cell lines	1,65E-02	BCL2A1,BCR,BLK,(	24	BL vs. M6
Cell Death and Survival	Apoptosis of leukocyte cell lines	4,56E-02	BCL2A1,BCR,BLK,(	32	BL vs. M6
Cell Cycle	Arrest in G1 phase	2,01E-02	BCR,DCN,FOXO1,(	8	BL vs. M6
Cell Cycle	Arrest in G1 phase of B-lymphocyte derived cell lines	6,35E-03	BCR,FOXO1,GRID:	6	BL vs. M6
Infectious Diseases	Bacterial Infections	2,86E-02	CASP1,CASP4,CSK	5	BL vs. M6
Cell Death and Survival	Cell death of B-lymphocyte derived cell lines	1,65E-02	BCL2A1,BCR,BLK,(	25	BL vs. M6
Cell Death and Survival	Cell death of leukocyte cell lines	1,53E-02	ANTXR1,BCL2A1,E	39	BL vs. M6
Cellular Movement, Hematological System Development and Func	Cell movement of naive lymphocytes	1,21E-02	BACH2,CCL20,CX(	6	BL vs. M6
Cellular Development, Cellular Growth and Proliferation, Hematolo	Cell proliferation of leukocyte cell lines	3,27E-02	ABL2,BCR,BLK,BTI	44	BL vs. M6
Cellular Development, Hematological System Development and Fu	Commitment of lymphocytes	6,35E-03	DTX1,EBF1,IL6,PA	6	BL vs. M6
Cell Death and Survival	Delay in apoptosis of phagocytes	1,59E-02	CASP1,CASP4,IL6,	5	BL vs. M6
Cellular Development, Cellular Growth and Proliferation, Embryon	Development of B lymphocytes	2,19E-02	ADAM10,AFF1,AK	28	BL vs. M6
Cellular Development, Cellular Growth and Proliferation, Embryon	Development of B-1 lymphocytes	1,71E-03	AKT2,BTK,EBF1,LY	7	BL vs. M6
Cellular Development, Cellular Growth and Proliferation, Hematolo	Development of bone marrow-derived macrophages	4,00E-02	AKAP13,CAV1,FO:	8	BL vs. M6
Cellular Development, Cellular Growth and Proliferation, Embryon	Development of follicular B lymphocytes	1,17E-02	ADAM10,AKT2,BT	7	BL vs. M6
Cellular Development, Cellular Growth and Proliferation, Embryon	Development of marginal-zone B lymphocytes	6,35E-03	ADAM10,AKT2,BT	6	BL vs. M6
Cellular Development, Cellular Growth and Proliferation, Embryon	Development of plasma cells	2,86E-02	BCL6,CXCL12,DICI	5	BL vs. M6
Cellular Development, Cellular Growth and Proliferation, Hematolo	Differentiation of myeloid progenitor cells	4,84E-02	BTK,FIP1L1,IL15,IL	6	BL vs. M6
Cellular Development, Cellular Growth and Proliferation, Embryon	Differentiation of naive lymphocytes	4,55E-02	AHR,BCL6,EFNB2,	12	BL vs. M6
Cellular Development, Cellular Growth and Proliferation, Embryon	Differentiation of pro-B lymphocytes	4,00E-02	BLK,EBF1,ETV6,LY	8	BL vs. M6
Cell-To-Cell Signaling and Interaction, Inflammatory Response	Immune response of leukocyte cell lines	4,84E-02	APP,CD38,FOXO1,	6	BL vs. M6
Cell Cycle	Interphase of leukocyte cell lines	1,35E-02	BCR,CXCL12,FOX(	8	BL vs. M6
Cell Death and Survival	Killing of leukocyte cell lines	4,00E-02	CD22,EXOC2,IL15,	6	BL vs. M6
Cellular Movement, Hematological System Development and Func	Migration of naive lymphocytes	7,63E-03	BACH2,CXCL12,C)	5	BL vs. M6
Hematological System Development and Function, Lymphoid Tissu	Morphology of lymph follicle	8,88E-03	BACH2,EPS15,FNI	6	BL vs. M6
Cellular Development, Cellular Growth and Proliferation, Connecti	Osteoclastogenesis of macrophages	4,42E-02	CAST,CAV1,DICER	9	BL vs. M6
Cellular Development, Cellular Growth and Proliferation, Hematolo	Osteoclastogenesis of phagocytes	4,17E-02	CAST,CAV1,DICER	10	BL vs. M6
Cell Morphology	Polarization of Th17 cells	2,86E-02	AHR,CD36,IL15,IL(	5	BL vs. M6
Hematological System Development and Function, Hypersensitivit	Quantity of mast cells	1,68E-02	BCL2A1,CASP1,CA	12	BL vs. M6
Hematological System Development and Function, Hematopoiesis,	Quantity of pre-B lymphocytes	2,30E-02	AFF1,AKAP13,ARI	24	BL vs. M6
Cell Signaling, Small Molecule Biochemistry	Synthesis of nitric oxide	3,61E-02	ALOX5,APP,BDNF,	17	BL vs. M6
Connective Tissue Disorders, Immunological Disease, Inflammator	Systemic juvenile idiopathic arthritis	2,81E-02	ASAP1,ATP2B1,CA	14	BL vs. M6
Gene Expression	Transactivation of RNA	1,59E-02	CD300LB,CD300LI	5	BL vs. M6
Cellular Movement, Hematological System Development and Func	Transendothelial migration of lymphocytes	4,07E-02	CAV1,CXCL12,ENI	7	BL vs. M6
Cellular Movement, Hematological System Development and Func	Transendothelial migration of mononuclear leukocytes	3,41E-02	CAV1,CCL7,CXCL1	8	BL vs. M6
Cancer	Transformation of B-lymphocyte derived cell lines	4,63E-02	ETV6,FIP1L1,HIP1	5	BL vs. M6
Cell-mediated Immune Response, Cellular Development, Cellular F	Transition of thymocytes	1,13E-02	BDNF,CHEK1,DOK	5	BL vs. M6
Cellular Function and Maintenance	Turnover of cells	2,16E-02	CCL20,CD22,DICEI	5	BL vs. M6
Cell Morphology, Humoral Immune Response, Immunological Dise	Abnormal morphology of B-1 lymphocytes	1,69E-02	CARD11,CR2,EBF1	5	M3 vs. M6
Hematological System Development and Function, Immune Cell Tr	Accumulation of regulatory T lymphocytes	3,21E-02	EGFR,IFNG,IKBKB,	6	M3 vs. M6
Cell-To-Cell Signaling and Interaction, Hematological System Devel	Activation of regulatory T lymphocytes	3,10E-02	BACH2,BCL11B,CL	9	M3 vs. M6
Cell Death and Survival	Apoptosis of B-lymphocyte derived cell lines	8,61E-03	ABL1,BCL2,BCL2L	27	M3 vs. M6
Cell Death and Survival	Apoptosis of leukocyte cell lines	1,05E-02	ABL1,BCL2,BCL2L	38	M3 vs. M6
Cell Death and Survival	Apoptosis of thymocytes	1,18E-02	ABL1,BBC3,BCL11	29	M3 vs. M6
Cell Cycle	Arrest in G1 phase of B-lymphocyte derived cell lines	4,17E-02	ABL1,BCR,GRID2,I	5	M3 vs. M6
Cell Death and Survival	Cell death of leukocyte cell lines	2,70E-02	ABL1,BCL2,BCL2L	41	M3 vs. M6
Cell Death and Survival	Cell death of monocyte-derived dendritic cells	1,43E-02	BCL2,BCL2L1,IKBK	6	M3 vs. M6
Cell Death and Survival	Cell death of thymocytes	2,11E-02	ABL1,BBC3,BCL11	31	M3 vs. M6
Cellular Development, Cellular Growth and Proliferation, Hematolo	Cell proliferation of leukocyte cell lines	2,26E-03	ABCA3,ABL1,ABL2	54	M3 vs. M6
Cell Death and Survival	Cell viability	2,43E-02	ABL1,ADGRE2,API	59	M3 vs. M6
Cell Death and Survival, Hematological System Development and F	Cell viability of B-lymphocyte derived cell lines	4,18E-02	ABL1,BCL2,BCL2L	14	M3 vs. M6
Cell Death and Survival, Hematological System Development and F	Cell viability of leukocyte cell lines	3,82E-02	ABL1,BCL2,BCL2L	17	M3 vs. M6
Cellular Function and Maintenance	Cellular homeostasis	4,10E-02	ABL1,ABL2,ADOR	126	M3 vs. M6
Cellular Development, Hematological System Development and Fu	Commitment of lymphocytes	1,03E-02	DTX1,EBF1,IL6,PA	6	M3 vs. M6
Cellular Development, Cellular Growth and Proliferation, Embryon	Development of B lymphocytes	2,57E-02	AFF1,BCL2,BCL2L	30	M3 vs. M6
Cellular Development, Cellular Growth and Proliferation, Embryon	Development of plasma cells	4,17E-02	CR2,DICER1,IL21R	5	M3 vs. M6
Cellular Development, Cellular Growth and Proliferation, Embryon	Development of pro-B lymphocytes	3,75E-02	BLK,FYN,IFNG,LYN	7	M3 vs. M6
Cellular Development, Cellular Growth and Proliferation, Embryon	Differentiation of pro-B lymphocytes	9,17E-03	BLK,EBF1,ETV6,FY	10	M3 vs. M6
Cellular Development, Cellular Growth and Proliferation, Hematolo	Expansion of hematopoietic progenitor cells	1,20E-02	BCL2L1,EBF1,EON	8	M3 vs. M6
Cell-mediated Immune Response, Cellular Development, Cellular F	Expansion of thymocytes	1,91E-02	EOMES,IKZF1,LCP	6	M3 vs. M6
Cellular Function and Maintenance	Lymphocyte homeostasis	2,49E-02	ABL1,ABL2,ADOR	114	M3 vs. M6
Antigen Presentation, Cell-To-Cell Signaling and Interaction, Cell-m	Positive selection of thymocytes	3,75E-02	CADM1,ELK4,NF1	7	M3 vs. M6
Cellular Development, Cellular Growth and Proliferation, Hematolo	Proliferation of hematopoietic progenitor cells	4,42E-02	BBC3,BCL2,BCL2L	25	M3 vs. M6
Cellular Development, Cellular Growth and Proliferation	Proliferation of myeloid cells	2,53E-02	CUX1,CYSLTR2,DC	23	M3 vs. M6
Cell-mediated Immune Response, Cellular Development, Cellular F	Proliferation of thymocytes	3,34E-02	BBC3,BLM,EGR3,E	18	M3 vs. M6
Hematological System Development and Function, Hypersensitivit	Quantity of mast cells	1,47E-02	ATF3,CASP1,CASP	13	M3 vs. M6
Cell Cycle	Replication of cells	1,43E-02	CASP1,IFNG,NLRP	6	M3 vs. M6
Connective Tissue Disorders, Immunological Disease, Inflammator	Systemic juvenile idiopathic arthritis	1,38E-02	ASAP1,ATP2B1,D(	16	M3 vs. M6
Cell-mediated Immune Response, Cellular Function and Maintenar	T cell homeostasis	4,33E-02	ABL1,ABL2,ADOR	106	M3 vs. M6
Cellular Function and Maintenance	Turnover of cells	3,19E-02	BCL2L1,CD22,DICI	5	M3 vs. M6

Supplementary Table 4. Functions affected by differentially methylated genes in CD4+ T-cells after treatment with DMF.

DMF: Dimethyl fumarate; BL: baseline; M3: 3 months after initation of DMF treatment; M6: 6 months after initation of DMF treatment.
Input gene list was based on genes associated with differentially methylated probes that displayed delta b > 5% methylation change and p-value < 0.001.
The table includes all functional annotations from Ingenuity Pathway Analysis with Fisher's p-value < 0.05 and number of molecules ≥ 5.

Supplementary Table 5. Upstream regulators predicted to explain a pattern of differentially methylated genes in CD4+ T-cells after treatment with DMF.

Upstream Regulator	Molecule Type	P-val	Target molecules in dataset	N mol	Group
NOTCH2	transcription regulator	7,64E-04	DTX1,IL22,IL6,TNF,TSPAN33	5	BL vs. M6
EP300	transcription regulator	2,64E-03	ACOXL,AHI1,AKAP12,ARHGAP24,BCL6,BTK,C	36	BL vs. M6
WNT3A	cytokine	2,83E-03	IL6,NANOG,RUNX1,RUNX1T1,TNF	5	BL vs. M6
STAT5B	transcription regulator	9,89E-03	ASAP1,BCL6,CASP4,CASP6,CASP8,CHEK1,EBF	16	BL vs. M6
CREBBP	transcription regulator	1,31E-02	ACOXL,AHI1,AKAP12,ARHGAP24,BCL6,CA2,C	34	BL vs. M6
GLI3	transcription regulator	2,21E-02	EBF1,FOXO1,IRF1,KLF13,PAX5,PTCH1,RUNX1	7	BL vs. M6
NOTCH1	transcription regulator	2,70E-02	ADAM19,ASB2,BCL6,IL22,IL6,TNF,TSPAN33	7	BL vs. M6
MAPK8	kinase	3,11E-02	BCL2,DUSP1,EOMES,IFNG,IL6	5	M3 vs. M6
IL17A	cytokine	3,26E-02	BCL2A1,CCL20,CD83,IL22,IL6,RGS13,TNF	7	BL vs. M6
VEGFA	growth factor	3,32E-02	CD1A,EOMES,IFNG,IL6,ITGAV,KLRG1,NFATC	8	M3 vs. M6
TCF3	transcription regulator	3,39E-02	CDC25B,CIP2A,FOXO1,SERINC5,TMEM184B	5	BL vs. M3
MED1	transcription regulator	3,55E-02	BCL11A,BCL6,EBF1,FOXO1,KLF13	5	BL vs. M6
NOTCH1	transcription regulator	4,40E-02	BCL2,BCL2L1,CFLAR,FLT1,IFNG,IL6,SMARCC1	7	M3 vs. M6
RORC	ligand-dependent nuclear receptor	4,65E-02	CCL20,IL1R1,IL22,IL4I1,IL6,TNF	6	BL vs. M6

Supplementary Table 5. Upstream regulators predicted to explain a pattern of differentially methylated genes in CD4+ T-cells after treatment with DMF.

DMF: Dimethyl fumarate; BL: baseline; M3: 3 months after initation of DMF treatment; M6: 6 months after initation of DMF treatment.
Input gene list was based on genes associated with differentially methylated probes that displayed delta b > 5% methylation change and p-value < 0.001.
The table includes all upstream regulators from Ingenuity Pathway Analysis with p-value of overlap < 0.05 and number of target molecules ≥ 5.

Supplementary Table 6. Plasma cytokine profile

	Responder			Responder			Non-responder			Non-responder			adj *P*-value Baseline	adj *P*-value 6months	adj *P*-value Responders	adj *P*-value Non-responders
Marker	Baseline	±SD	n	6months	±SD	n	Baseline	±SD	n	6months	±SD	n	Resp/non-Resp	Resp/non-Resp	Baseline/6m	Baseline/6m
IL17C	0,536	0,625	29	0,365	0,497	29	0,861	0,565	11	0,397	0,474	11	-	-	0.0011	-
IL17A	0,235	0,333	29	0,182	0,346	29	0,260	0,257	11	0,079	0,176	11	-	-	-	-
CCL28	0,889	0,223	29	0,084	0,397	29	1,171	0,430	11	1,166	0,358	11	-	<0.0001	<0.0001	-
CDCP1	1,654	0,549	29	1,874	0,519	29	1,563	0,396	11	1,971	0,599	11	-	-	-	-
EN-RAGE	1,546	0,485	29	1,148	0,537	29	1,419	0,569	11	1,134	0,331	11	-	-	-	-
SLAM	1,699	0,316	29	1,508	0,495	29	1,691	0,318	11	1,690	0,356	11	-	-	-	-
IL12B	4,628	0,759	29	5,130	0,662	29	4,784	0,491	11	5,100	0,364	11	-	-	0.0081	-
IL18R	6,841	0,496	29	6,696	0,426	29	6,802	0,281	11	6,891	0,279	11	-	-	-	-
CXCL9	5,944	0,973	29	6,500	0,917	29	5,807	0,730	11	6,214	1,001	11	-	-	0.0026	-
CCL4	6,229	0,695	29	6,459	0,608	29	6,198	0,501	11	6,364	0,584	11	-	-	-	-
TWEAK	8,567	0,328	29	8,407	0,307	29	8,701	0,377	11	8,550	0,319	11	-	-	-	-

Supplementary Table 6. Plasma cytokine profile

The table provides statistics (student t-test/ paird t-test) in cytokine levels
Additional Olink information could be found at https://www.olink.com/
